# An eczematous eruption with features of prurigo nodularis: An unexpected presentation of alpha-gal syndrome

**DOI:** 10.1016/j.jdcr.2025.01.033

**Published:** 2025-03-01

**Authors:** Shaun Wu, Emily R. Nadelmann, Merav Koschitzky, Yevgeniy Balagula, Caroline Halverstam, Tobi Klar

**Affiliations:** Department of Dermatology, Jacobi Medical Center, Albert Einstein College of Medicine, Bronx, New York

**Keywords:** AGS, alpha-gal syndrome, anaphylaxis, angioedema, eczematous rash, galactose-alpha-1,3-galactose, gastrointestinal disturbances, IgE-mediated allergy, mammalian meat allergy, prurigo nodularis, red meat allergy, tick-borne allergy, urticaria

## Introduction

Alpha-gal syndrome (AGS) is an immunoglobulin E-mediated hypersensitivity reaction against galactose-alpha-1,3-galactose (alpha-gal), a carbohydrate found in red meat and other animal products.^1^ Common alpha-gal containing foods include red meat such as beef, pork, lamb, and deer, diary such as milk, cheese, yogurt, and butter, and gelatin such as marshmallow, jelly babies, and other sweets.^2^ This condition is induced by tick bites, which sensitize individuals to alpha-gal in tick saliva.^3^

Clinical features of AGS are diverse and include urticaria, angioedema, and gastrointestinal disturbances manifesting as severe stomach pain. Anaphylaxis, a severe life-threatening reaction, is also fairly frequent in AGS patients.^4^ Symptom severity and tolerance to red meat can vary among patients. Thus, this condition can be difficult to diagnose, and many cases are often misdiagnosed as anaphylaxis or urticaria. The average time to diagnosis is 7 years.^5^^,^^6^

Although an urticarial eruption is the hallmark cutaneous manifestation in AGS, we present a case of a patient with an eczematous rash featuring prurigo nodularis, which illustrates the variability in clinical presentations. This atypical presentation points to the need for an increase in clinical awareness and consideration of AGS in patients presenting with dermatological symptoms that are atypical following consumption of mammalian meat.

## Case report

**Fig 1:**
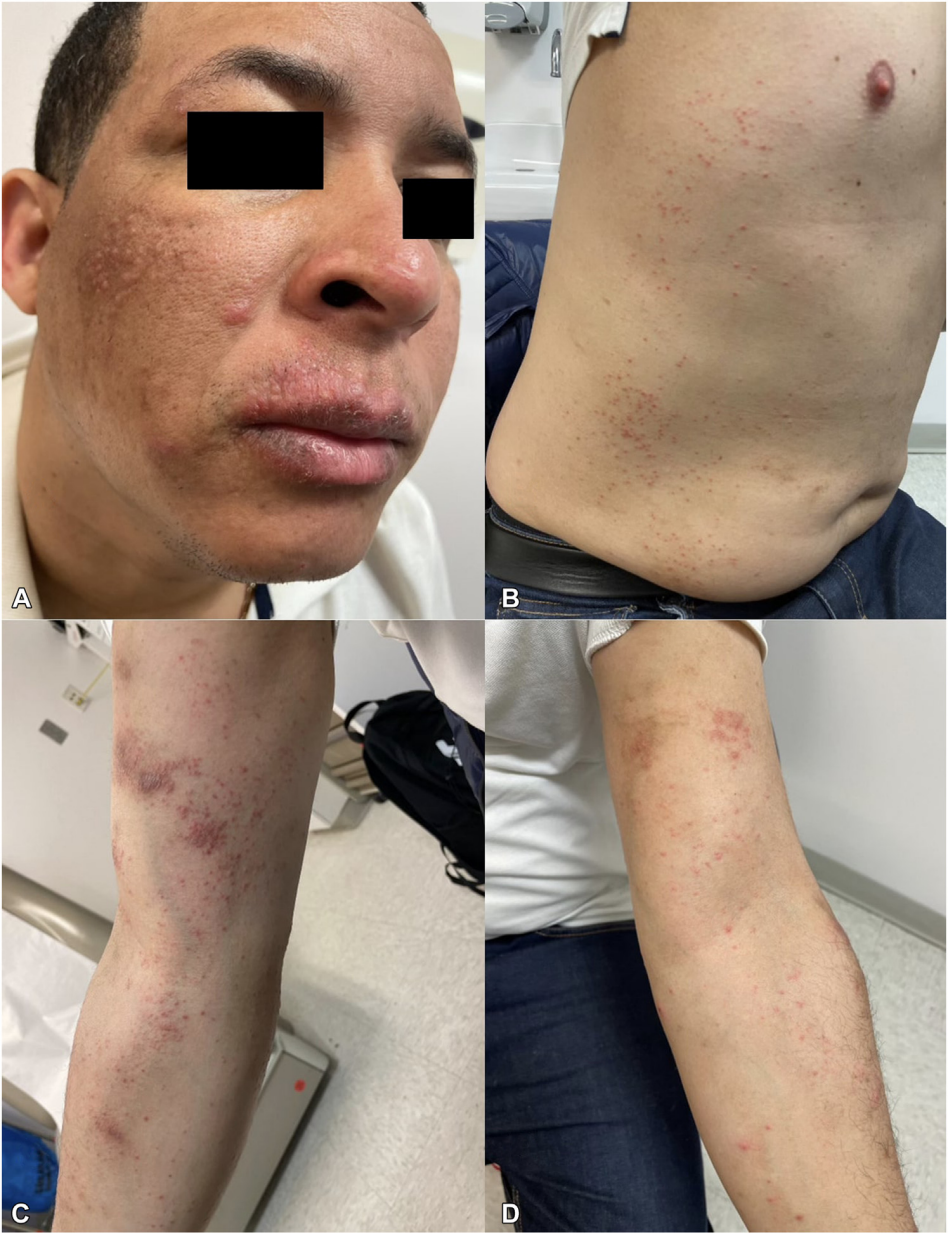
Clinical presentation (**A**) erythematous edematous papules of upper lips and cheeks (**B)** Multiple scattered small, erythematous papules of trunk (**C** and **D**) erythematous to violaceous eczematous papules of bilateral arms.

A 34-year-old male presented to our clinic with a 2 year history of a recurrent pruritic rash on his face, bilateral arms, and trunk (Fig 1, *A*-*D*). The rash, which initially appeared on one arm, gradually spread to both arms before eventually covering the entire body, sparing only the back. The rash, especially severe on his face and lips, sometimes caused bleeding. He found minimal relief with over-the-counter hydrocortisone cream. He denied any new triggers, including history of new medications, fragrances, recent hiking, travel, sick contacts, or changes in bedding. He was diagnosed with an eczematous reaction given the morphology of his rash, and was treated with a high-potency topical corticosteroid for prurigo nodularis.

Over the following several months, however, these symptoms continued despite the addition of intralesional kenalog injections into prurigo lesions and an intramuscular kenalog injection. Given the persistence of his symptoms, a punch biopsy was performed, which demonstrated a spongiotic dermatitis with eosinophils consistent with an eczematous eruption.

It was not until a follow-up appointment the following year that he noted lip swelling with the ingestion of a red apple, which resolved spontaneously following treatment with diphenhydramine. He later recalled experiencing lip edema in the past, following ingestion of red meat. Upon further questioning, the patient mentioned a history of a tick bite after hiking several years earlier. AGS was then considered given the history of the angioedema upon red meat ingestion. Lab work-up subsequently revealed an elevated specific serum alpha-gal immunoglobulin E level of 1.12 kU/L (Normal ≤0.1 kU/L).^5^ The patient was advised to avoid red meat and alpha-gal-containing products, provided with an epinephrine auto-injector, and was referred to allergy/immunology. Follow-up appointments and further testing were scheduled to monitor progress and response to treatment. After eliminating meat, diary, gelatin, and other foods containing alpha-gal, and initiating Dupixent, his skin has shown marked improvement, with significant resolution of his lesions.

## Discussion

Current management of AGS involves strict avoidance of mammalian meat and alpha-gal-containing products, as well as measures to prevent tick bites. Patients may be prescribed antihistamines, oral cromolyn solution, oral corticosteroid, omalizumab, and metformin. In some cases, patients on omalizumab or metformin have even been able to return a small amount of red meat in their diets.^5^ Intramuscular epinephrine are typically prescribed for patients with AGS to use in case they develop severe reactions such as anaphylaxis.^7^

Diagnosing AGS can be challenging, as patients are frequently misdiagnosed, and the correct diagnosis may take years.^8^ Distinguishing AGS from other conditions is often difficult. In our case, the patient was initially diagnosed with a nonspecific eczematous eruption with prurigo nodularis. His symptoms were intermittent, varying in severity from a mild pruritic rash on his trunk and extremities to severe pruritus and lip swelling, leading to excoriations and bleeding. This suggests that the severity of allergic reaction in AGS may vary with each exposure to triggers. The unpredictable nature of symptoms may in itself serve as a diagnostic criteria for AGS.^5^

Multiple studies describe urticaria, angioedema, and pruritus as the primary cutaneous reactions to ingestion of mammalian meat, with 80% to 90% of AGS patients experiencing these symptoms.^9^ However, there are a few instances where patients exhibited other cutaneous symptoms including purpuric rash, psoriasiform rash, nummular eczema, and subcutaneous nodules.^6^ These cases highlight that while urticaria, pruritus, and angioedema are the most common symptoms, AGS can also manifest with various other cutaneous reactions.

Diagnosing AGS in our patient was particularly difficult because he did not initially report any history of tick bites and denied any particular triggers. It was not until a follow-up visit a few years later that our patient recalled a tick bite and noted angioedema following red meat ingestion. This highlights the importance of considering AGS as a potential diagnosis even if the patient does not initially report tick bites or reactions to red meat. Tick bites or reactions to red meat may be too subtle for the patient to notice initially, or the connection between red meat ingestion and symptoms may not be immediately apparent. Both can lead to a significant delay in the diagnosis. Early consideration of AGS can invoke prompt laboratory testing and diagnosis, significantly improving the patient’s quality of life.

## Conflicts of interest

None disclosed.
